# Distress Behavior Conversations: Supporting Whole Person Whole Team Responses in VA Community Living Centers

**DOI:** 10.1093/geroni/igab046.3095

**Published:** 2021-12-17

**Authors:** Julia Loup, Kate Smith, Susan Wehry, Sharon Sloup, Jennie Keleher, Princess Nash, Christine Hartmann, Andrea Snow

**Affiliations:** 1 University of Alabama, Tuscaloosa, Alabama, United States; 2 University of New England College of Osteopathic Medicine, Biddeford, Maine, United States; 3 Tuscaloosa VA Medical Center, Tuscaloosa, Alabama, United States; 4 VA Bedford Healthcare System, VA Bedford Healthcare System, Massachusetts, United States

## Abstract

Resident distress behavior, a prevalent challenge in long-term care, contributes to resident morbidity, staff burden, and turnover. We describe an education model developed in the Veterans Administration (VA) Community Living Centers (CLC) through a CONCERT (VA CLCs’ Ongoing Center for Enhancing Resources & Training) quality improvement series. The Distress Behavior Conversation (DBC) uses a team meeting structure and process. Informed by unmet need and relational coordination theories, it guides the whole team, inclusive of interdisciplinary team members and front-line staff with resident contact, through a collaborative problem-solving action-planning discussion. DBC uses facilitated round-robins to identify potential resident behavior causes and individualized solutions. DBC supports the team in maintaining whole person and whole team mindsets, thus challenging the narrower medical model of discipline-specific clinical mindsets and staff level hierarchies. Over two years we have co-created and refined DBC through trainings and team debriefings with over 80 CLCs. Care teams reported “aha” moments during DBCs their thinking shifted (“we are now looking at the REAL why”; “we went from asking, how did he fall? to, why did he fall?; “tended to try to treat falls in a standardized way, [but] when you focus on a specific person you get to focus on HIS needs”; “personal information about the Veteran is the 5th vital sign!”). Teams additionally reported reduced strain and improved collaborative thinking (“I feel better about what I’m doing...more motivated to keep going!; “Now I see it is a team approach – don’t have to do it by myself.”).

